# ‘Matrioska left ventricle’: a case of double-chambered left ventricle with double mitral valve apparatus

**DOI:** 10.1093/ehjci/jeac072

**Published:** 2022-05-03

**Authors:** Chiara Rovera, Federica Valli, Alessandra Volpe, Marco Guazzi, Gianluca Pontone

**Affiliations:** Centro Cardiologico Monzino IRCCS, Via Carlo Parea, 4, 20138 Milan, Italy; Cardiology Division, Department of Health Sciences, ASST Santi Paolo e Carlo, Via Antonio di Rudinì, 8, 20142 Milan, Italy; Centro Cardiologico Monzino IRCCS, Via Carlo Parea, 4, 20138 Milan, Italy; Cardiology Division, Department of Health Sciences, ASST Santi Paolo e Carlo, Via Antonio di Rudinì, 8, 20142 Milan, Italy; Centro Cardiologico Monzino IRCCS, Via Carlo Parea, 4, 20138 Milan, Italy

A 37-year-old man was hospitalized in June 2021 for SARS-CoV2 pneumonia. Electrocardiogram showed repolarization abnormalities, suggestive of hypertrophic cardiomyopathy (HCM). Two-dimensional transthoracic echocardiography demonstrated hypertrophic muscle strands in the mid-apical portion of the left ventricle (LV) with increased pressure gradient at the level of mid-ventricular narrowing, this one is well depicted with the 3D method (upper panels and see Supplementary data online, *Movies S1–S3*). Global LV systolic function was mildly reduced. Genetic testing for HCM was negative. Cardiac magnetic resonance (middle panels and see Supplementary data online, *Movies S4* and *S5*) and computed tomography (lower panels and see Supplementary data online, *Movie S6*) demonstrated that LV was divided into two chambers by an anomalous fibromuscular septum. This bundle had a synchronous movement with the LV wall and was mimicking the systo-diastolic fluctuation of the mitral valve leaflets. Papillary muscles and trabeculae were organized in a confluent manner in order to recreate a sort of mitral valve duplicate at the mid-LV level.

**Figure:**
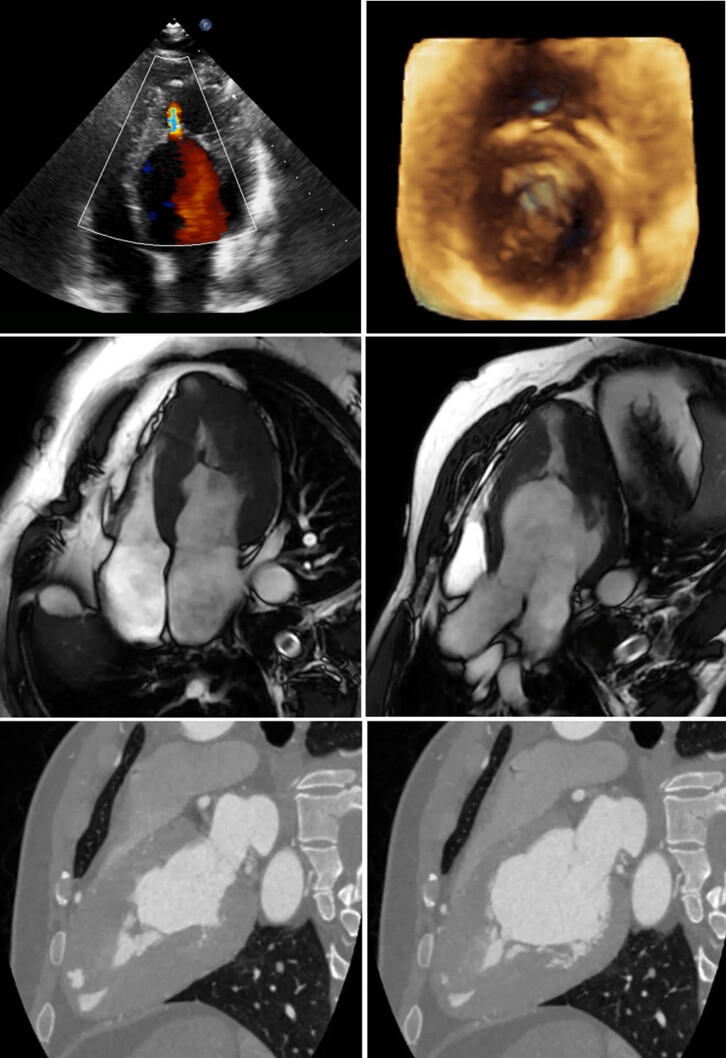


Double-chambered left ventricle (DCLV) is a very rare congenital cardiac anomaly. A genetic heterogeneity was found. Type A has a superior–inferior arrangement, related to the position of the main and accessory chambers, and sometimes presents intraventricular obstruction. DCLV is usually asymptomatic. The time of occurrence and the degree of clinical symptoms are linked with the size of the orifice between the two chambers and the LV function. To our knowledge, this is the first case of DCLV with associated well-defined double mitral valve apparatus at the mid-LV level. Multimodality cardiac imaging is of pivotal importance for confirming the diagnosis of DCLV.

Supplementary data are available at *European Heart Journal – Cardiovascular Imaging* online.

**Conflict of interest:** None declared.

**Funding:** None declared.

**Data availability** Data available within the article and its supplementary materials.

## Supplementary Material

jeac072_Supplementary_Data

